# Travelling the Last Mile – Bringing Evidence to Individuals in Israel: a commentary on building capacity in implementation science

**DOI:** 10.1186/s13584-025-00705-4

**Published:** 2025-07-10

**Authors:** Kenneth J. Mukamal, Lital Keinan-Boker

**Affiliations:** 1 Department of Medicine, Beth Israel Deaconess Medical Center, Boston, MA USA; 2 Faculty of Social Welfare & Health Sciences, School of Public Health, University of Haifa, Haifa, Israel

**Keywords:** Implementation science, Generalizability, Quality improvement, Epidemiology

## Abstract

In their previously published article in the Israel Journal of Health Policy Research, Rose and colleagues describe and advocate for greater use of implementation science in Israel. As a discipline, implementation science seeks to traverse the last steps in bringing new science from research to clinical practice, which are often the most difficult of the entire process. Implementation science in general faces substantial challenges, including the extraordinary heterogeneity of the dissemination process, and the obstacles represented by established practices, singular preferences, and questions about generalizability. In our view, implementation science complements classic epidemiology as part of a continuum of population health research that warrants greater attention and funding. For now, however, implementation science will need to show that it can consistently achieve sizable, durable, and widespread results if it is to traverse its own last mile and establish itself as a successful and permanent component of biomedicine in Israel.

For the last four decades, as telecommunications firms have sought to bring ever greater volumes of electronic data to individual homes, one axiom has held true—the last mile is the hardest. The process of moving large tranches of data through major electronic hubs, while challenging, is nonetheless technically reproducible and standardized; it is, in essence, the electronic arterial tree and (in the absence of atherosclerosis) flows quickly and reliably. In contrast, when distributing much smaller batches of data to multiple individual households, local geography, regulation, and competing infrastructure can forestall even the most herculean telecommunication engineering efforts. This terminal leg of the data journey is its capillary system, which in humans exceeds 6,000 m^2^ in area—a simply astonishing requirement for bringing oxygen its last few microns that illustrates the magnitude of this challenge.

As previously published in the Israel Journal of Health Policy Research [1], Rose and colleagues describe and advocate for greater use of implementation science in Israel. As they note, implementation science seeks to identify effective (and ideally cost-effective) ways to implement ‘proven’ science, particularly in clinical settings. It is, essentially, the practice of studying and traversing those difficult last few kilometers, where scientific advancement has already occurred but not yet reliably filtered down to individual patients, providers, and clinics. As the telecommunications and vascular examples make clear, these last steps are often the most difficult of the entire process. In this editorial, we first highlight some of the general challenges faced by implementation science and then address the authors’ specific recommendations for expansion of implementation science in Israel.

## General issues facing implementation science

In an ideal world, implementation scientists can identify general strategies that may be useful across multiple settings and potentially for diverse interventions. In reality, however, the specifics of individual circumstances introduce extraordinary heterogeneity into the dissemination process, and established practices, singular preferences, and legitimate questions about generalizability may all obstruct the introduction of new paradigms. For all these reasons, implementation warrants careful and dedicated attention for success.

How do established practice, singular preference, and generalizability manifest in the last-mile problem? When important new advances in clinical care or public health occur, they appear first in peer-reviewed scientific journals (like IJHPR) as efficacy studies, followed ideally by tests of effectiveness in practical settings, and then in review articles and society guidelines. At that point, health systems might legitimately seek to introduce the new practices into everyday care. The progressive lowering of systolic blood pressure treatment targets for essential hypertension presents a cogent example of such an innovation. Once SPRINT demonstrated a profound mortality benefit for individuals randomized to intensive blood pressure treatment [2], even among older participants [3], guidelines gradually recognized the need to set lower targets for therapy [4]. At that point, it became incumbent upon health systems and individual providers to find ways to meet these updated targets; failure to meet the updated standard of care could put them at legal and financial risk. However, substantial challenges have continued to thwart the goal of widespread tight blood pressure control; indeed, guidelines on implementation strategies appeared almost immediately after the original hypertension treatment guidelines in recognition of this challenge [5]. How do these challenges manifest? First, new goals require upending established practice patterns, which themselves often required explicit efforts to attain. This iterative process of advancement can leave clinicians understandably frustrated and cynical as targets appear ever further out of reach. Clinicians must also overcome patient reluctance to intensify therapy (with no apparent immediate benefit) and their own therapeutic inertia [6], all while disrupting establishing care pathways. Superimposed on this more widespread impediment, patients and providers can continue to make individualized decisions about goals and preferences for care. Put differently, each clinical interaction represents an opportunity for patients and providers to set their own prior probabilities for the likelihood of benefit or harm, introducing inevitable heterogeneity in the pursuit of patient-centered care. Finally, legitimate concerns exist about whether trials like SPRINT are truly generalizable (even when guideline writers have concluded that they are) [7]. These concerns, whether proven by evidence or not, may amplify hesitancy to change existing practice. Achieving universal adoption of any new therapy, no matter how beneficial, is probably impossible.

It is reasonable to ask how implementation currently occurs for new and potentially challenging interventions in other contexts. As the authors note, important developments in and outside of health care will frequently come from outside Israel, an inevitability given its relative youth, limited size, and substantial immigrant population. The US poses a particularly interesting contrast in this regard, for it has no discernible ‘health care system’ but rather a heterogeneous collection of independent health care practitioners grouped into practices of wildly different size, scope, and investment. These may be privately owned, run as not-for-profit, government funded, or, increasingly, administered by private equity firms. As a consequence, it has proven extraordinarily difficult to identify approaches that can truly be implemented on a wide scale. To a sizable degree, this has practically meant that systems and organizations must individualize their methods, most commonly when faced with a financial incentive to do so. For example, with funding from the Centers for Medicare and Medicaid Services (CMS), the National Committee for Quality Assurance (NCQA) publishes its Healthcare Effectiveness Data and Information Set (HEDIS), a set of indicators that health insurers across the US use to measure the quality of care delivered by physicians and health systems. Because of their associated financial rewards and penalties, they receive disproportionate attention and, in turn, spawn numerous (but largely unlinked) quality improvement efforts nationwide. Given the financial incentives involved, the size, scope, and effectiveness of such efforts can be impressive indeed [8]. With enough time and sufficiently strong financial incentives, these can achieve substantial success, sometimes so widespread that HEDIS indicators can be retired when they achieve saturation [9].

Rose and colleagues attempt to differentiate implementation science from the more common and recognizable domain of quality improvement, largely based on the former’s larger size and scope. In our view, this represents distinction without difference; many self-described quality improvement initiatives are large, rigorous, and impactful [10]. Indeed, Israeli efforts at quality improvement, such as early repair of hip fracture, have clearly influenced clinical practice, even if their long-term impact may be delayed [11]. Regardless of this nomenclature, however, implementation sciences stands at an important cusp relative to traditional clinical and basic disciplines—a ledge in which it could be relegated to an ad hoc approach to addressing problems as they arise or gain traction as an attractive form of inquiry. In our view, this will ultimately depend on the degree that implementation science can navigate the tension between generalizability and practicality. Biomedical investigators rightly assume that human physiology does not differ meaningfully between Boston and Tel Aviv, but even the most fervent implementation scientists would be challenged to find strategies that can be employed successfully to, say, maximize vaccination rates or ensure statin use among diabetic adults in those two contexts. Moreover, the costs of failing to enact successful implementation strategies (in terms of failing to meet quality guidelines or stay within budget) are generally local, so that little incentive or opportunity may exist to develop, test, and execute implementation programs that have a truly national scope and exhibit robust generalizability. Put differently, the strongest drivers of successful implementation are often located at the local or regional levels, resulting in programs that have local or regional utility, but with no easy or guaranteed mechanism to develop large-scale, reliable programs. Unfortunately, even the examples that Rose and colleagues cite illustrate this challenge. For example, they highlight the STORM prediction model for opioid-related adverse events, but this is not even the only federally recommended model in the US, dozens of other private tools have been proposed, and the tool itself was intentionally designed for use in the restricted population of veterans [12, 13]. For implementation science to thrive in Israel as Rose and colleagues envision, it will have to demonstrate scalability at least at the national level and ideally also when applied to hard-to-reach populations (e.g., among individuals with lower socioeconomic status or non-Jews).

## Expanding implementation science in Israel

Rose and colleagues raise several questions and make several recommendations regarding implementation science in Israel. Although the authors provide an interesting and, in many respects, compelling case for implementation science, several factors color the feasibility and expected utility of their suggestions.

For example, the authors describe epidemiology as “unlikely to produce health system changes,” although, as a quantitative methodology, epidemiology is not intended to produce change strictly on its own. The authors then frame epidemiology as prominent and amply funded in Israel, despite its ostensible inability to change health systems. In fact, the largest research funders in Israel, such as the Israeli Science Foundation, often prioritize basic science projects. Similarly, the National Institute for Health Policy Research, which is funded by the National Health Insurance Law, eschews purely epidemiological studies that are not directly associated with health policy. As a result, the authors’ apposition of implementation science against epidemiology neglects the reality that this entire continuum of population health research warrants greater funding.

The authors appropriately note that Efshari Bari, an Inter-Ministerial (Health, Education, Sports) health promotion intervention, did not undergo formal evaluation. Interestingly, the program included a designated component of evaluation as originally designed, but budget cuts halted the program before its evaluation, and when re-commenced, that component was not restored. This sequence of events highlights how limited budgets impose inevitable trade-offs that may jeopardize the role of implementation science; by very definition, completing the last mile requires completion of all earlier miles first.

One factor in Israel’s favor with respect to its ability to achieve full implementation of important public health initiatives is its demonstrated success in confirming real-world effectiveness using health care records and national registries. In most situations worldwide, only efficacy testing is required for approval, ignoring practical effectiveness and slowing the entire sequence that eventually leads to implementation. However, Israel has already demonstrated its ability to go beyond tightly controlled research settings. For example, the Israel Center for Disease Control in the Ministry of Health has been particularly successful in producing real-world effectiveness with respect to vaccination efficacy [14, 15].

Finally, the authors proposed five formal recommendations for policy change in Israel. The first calls for schools of public health, medicine and nursing in Israel to abandon their “near exclusive emphasis on epidemiology” and actively seek to hire experts in implementation science and program evaluation. Unfortunately, this framing pits epidemiology as an "enemy" to be replaced by better options. To us, implementation science is an unquestionably valuable addition to the curriculum, and thus will indeed require investment in those who can teach and execute it. Ideally, it would become a core concept in the curriculum, alongside (but not in place of) classic epidemiological methods. However, at this point, we cannot endorse a formal degree in implementation science; such specialized programs remain very rare worldwide and hence need to be developed and vetted before stand-alone options can be firmly recommended. Moreover, successful implementation projects require diverse parties with complementary skills that include content knowledge, project design, project management, human factor engineering, data collection, data analysis, and even marketing and psychology; as such, we would encourage students to pursue additional training in implementation science in conjunction with, rather than in lieu of, their programs of study in these other focus areas.

The authors’ second call is for Israeli funders to gain familiarity with implementation science and ideally to dedicate funds for it. Unfortunately, funding options for epidemiological studies in Israel are already limited in size and scope, and thus creating a dedicated stream of funding for implementation science could jeopardize much of the population health research that undergirds it. The National Institute for Health Policy Research is a natural candidate for funding implementation projects, and we advocate designating part of its research budget for that purpose.

We generally agree with the third, fourth, and fifth recommendations, but would add that better transparency of health data, while desirable for evaluation purposes, must nonetheless resolve key privacy issues. Because implementation science generally studies individuals outside of a research context, its subjects have generally not given specific consent for those studies. In that setting, the pendulum must tilt firmly towards confidentiality and anonymity, and indeed Israel’s privacy protection regulations generally exceed those in the US. We would also highlight the database access that the Ministry of Health offer to investigators through the Timna platform. These data rely upon national registers (including births, immunizations, infant clinics, disease registries, hospitalizations, emergency room visits, and deaths). While not as expansive as primary health records, they offer outstanding opportunities for implementation science.

The authors’ proposal to bolster implementation science is bold and forthright. They rightfully state the case that implementation, without concerted efforts, is all-too-prone to fail. But implementation science itself will need to show that it can consistently achieve sizable, durable, and widespread results if it is to match other disciplines in notoriety and funding. Even for implementation scientists, the last mile is truly the hardest.

## Data Availability

No datasets were generated or analysed during the current study.
